# Fabrication of Composite Membrane by Constructing Helical Carbon Nanotubes in Ceramic Support Channels for Efficient Emulsion Separation

**DOI:** 10.3390/membranes15050150

**Published:** 2025-05-15

**Authors:** Kai Yuan, Rizhi Chen, Yiqing Zeng

**Affiliations:** 1 School of Art, Jiangsu Open University (The City Vocational College of Jiangsu), Nanjing 210036, China; 2 State Key Laboratory of Materials-Oriented Chemical Engineering, National Engineering Research Center for Special Separation Membrane, Nanjing Tech University, Nanjing 211816, China; rizhichen@njtech.edu.cn; 3 NJTECH University Suzhou Future Membrane Technology Innovation Center, Suzhou 215300, China; 4 School of Environmental Science and Engineering, Nanjing Tech University, Nanjing 211816, China

**Keywords:** helical carbon nanotubes (HCNTs), ceramic composite membrane, oil–water separation, membrane fouling, chemical vapor deposition (CVD)

## Abstract

Membrane technology has emerged as an effective solution for the purification of oily wastewater, particularly in the separation of oil-in-water (O/W) emulsions. However, challenges, such as membrane fouling and the development of robust ceramic membranes with superior stability, continue to limit their widespread application. In this work, helical carbon nanotubes (HCNTs) with interlocking structures were grown in ceramic support channels through the airflow-induced chemical vapor deposition (CVD) method to fabricate membrane material with high hydrophilicity and underwater oleophobicity. The influence of CVD parameters on the growth of HCNTs and the membrane separation performance for O/W emulsions were studied systematically. The optimal HCNTs-SiC composite membrane was prepared at 600 °C, featuring a pore size of 0.95 μm and flux of 229.29 L·m^−2^·h^−1^. This membrane demonstrated exceptional purification efficiency (99.99%) for a 500 ppm O/W emulsion, along with a stable flux of 32.48 L·m^−2^·h^−1^ under a transmembrane pressure (TMP) of 1.5 bar. Furthermore, the unique membrane structure and surface heterogeneity contributed to its long service life and excellent recovery capability. This work provides a novel strategy for designing high-performance ceramic membranes for oil–water separation, offering potential solutions to current limitations in membrane technology.

## 1. Introduction

With the rapid growth of the global population and accelerated industrialization, sustainable water resource management has emerged as a critical global challenge [1,2]. The increasing generation of oily wastewater [3] from diverse sources [4], including offshore oil spills [5], industrial discharges [6], and domestic activities [7] (e.g., cosmetics and catering industries), has not only contaminated clean water sources but also posed severe environmental threats and impeded sustainable human development [8]. Among various oil–water systems [9], surfactant-stabilized oil-in-water (O/W) emulsions, particularly those with small droplet sizes, present the most challenging separation problems [10]. Membrane separation technology [11,12,13] has gained significant attention for treating complex O/W emulsions due to its superior cost-effectiveness and separation efficiency compared to conventional methods [14], such as chemical flocculation [15] and biological processes [16]. While ceramic membranes [17,18,19] demonstrate exceptional advantages over polymeric membranes in terms of mechanical strength, chemical resistance, and thermal stability, their widespread application remains limited by persistent challenges, including membrane fouling [20,21,22] and low permeability [23,24,25]. Consequently, there is an urgent need to develop advanced membrane materials that simultaneously offer high separation efficiency, excellent antifouling properties, and facile regeneration capabilities.

Scholars have already constructed alternative options for separating O/W components [26]. For example, Gao [27] successfully constructed WPVDF nanofiber membranes through electrospinning technology, whose separation efficiency and permeation flux were >98.5% and 12,940.1 L·m^−2^·h^−1^ towards O/W emulsions. Liu [28] fabricated superhydrophobic/superoleophobic membrane (PAN@Co-MOF) and found that the flux surpassed 1600 L·m^−2^·h^−1^. Although these materials exhibit extremely high flux and good separation efficiency, these nanofibers still have significant disadvantages, such as poor thermal stability, unsatisfactory mechanical strength, and average resistance to acid and alkali corrosion. Fortunately, special nanofibers with excellent performance, carbon nanotubes (CNTs), have been discovered, and they exhibit great potential for oil–water separation. For example, Li [29] et al. deposited CNTs on stainless steel mesh through CVD to prepare a stain-resistant oil–water separation membrane. Dong [30] et al. constructed a CNT membrane layer on the surface of mullite support through the same method to prepare a composite separation membrane with easy regeneration and high-temperature-resistant performance. Those studies have shown that the composite membrane materials constructed with CNTs and ceramic supports exhibit excellent oil–water separation performance and regeneration capabilities. However, two challenges still need to be overcome. On the one hand, it is difficult for ceramic supports to form a strong bonding force with CNT membrane layers, which grow on the surface of ceramic supports. That leads to the risk of CNTs falling off from supports with high-rate liquid during the filtration or cleaning process, reducing the service life and performance of the membranes. On the other hand, for O/W emulsions, water with larger density could build a dynamic barrier on the membrane surface during the filtration process, preventing oil from permeating the membrane [31]. Therefore, the hydrophilic–underwater–oleophobic membrane material is more suitable for the separation of those emulsion systems, and it has the advantage of larger flux and better pollution resistance [32,33,34]. Growing CNTs with strong bonding force on ceramic support and constructing hydrophilic surfaces is expected to solve the technical bottleneck mentioned above.

Helical carbon nanotubes (HCNTs) [35,36,37], a new material discovered in recent years, have shown excellent mechanical properties and superior stretchability compared with the typical straight CNTs. When they are assembled to membrane materials, CNTs can physically intertwine/entangle/interlock with each other, which would enhance the strength and the integrity of the membrane [38,39,40]. Poli [41] et al. successfully grew CNTs in the pore channels of a 60 μm active layer prepared using 1 μm SiC particles for oil–water separation. The synthesized CNTs anchored well with the ceramic and would not fall off after several 30 min sonication cycles. Moreover, Li [42] et al. successfully synthesized hydrophilic helical carbon fiber using acetylene (C_2_H_2_) and Ni foam in one step via CVD. Consequently, the growth of HCNTs in ceramics is promising for the preparation of high-performance oil-in-water separation membranes, but, unfortunately, no relevant studies have been reported.

Herein, a novel strategy for fabricating an excellent emulsion separation composite membrane by growing HCNTs within SiC support channels via an airflow-induced CVD method was proposed. The HCNTs played three important roles: (1) effectively modifying the pore channels of the support body, thereby reducing the original pore size of tens of microns by an order of magnitude; (2) helical carbon nanotubes growing in the stacked pore structure resisted falling from the support; and (3) forming a heterogeneous surface to improve membrane antifouling. The optimized HCNTs-SiC composite membrane demonstrated high separation performance of 99.99% for O/W emulsions and recovery capability. This work not only provides a new approach for designing robust ceramic membranes for oil–water separation but also offers potential solutions to overcome the current limitations of membrane technology.

## 2. Materials and Methods

### 2.1. Experimental Materials

SiC powders with particle sizes of 200 μm (purchased from Yuda SiC powder Co., Ltd., Nantong, China) and activated carbon with a particle size of 300 nm (Shanghai Haino Carbon Industry Co., Ltd., Shanghai, China), as well as the sintering aids, such as mullite powder, calcium oxide, and zirconia (Weixi new material Co. Ltd., Changsha, China), were used as the main support raw material. Ethanol (C_2_H_5_OH, purity ≥ 97%) (Yasheng Chemical Co., Ltd., Wuxi, China) and ferrocene (purity ≥ 99%) (Aladdin Industrial Corporation Shanghai, China) were used as reactants to grow HCNTs. Nitrogen (purity ≥ 99%) (Nanjing Zhijun Special Gas Co., Ltd., Nanjing, China) was used as the shielding gas. Lubricant oil (Zhejiang Shell Chemical Petroleum Co., Ltd., Pinghu, China) and Span 80 (1/10 mass of the oil phase) (Linyi Unite Bio-technology Co., Ltd., Linyi, China) were used to make oil-in-water emulsions.

### 2.2. Fabrication of HCNTs-SiC Separation Membrane

**Figure 1:**
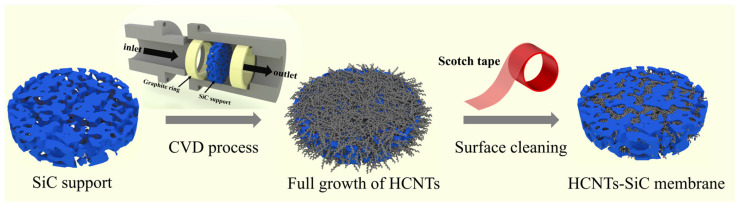
The schematic diagram for the preparation of the HCNTs-SiC composite membrane.

The preparation process for the ceramic SiC support was described in our previous work [43]. The porous SiC support was sandwiched with two graphite rings (in Figure 1) and placed into a stainless reactor consisting of concave and convex chimeric structures. The reactor was then placed into the heating furnace after checking air tightness. We prepared a 50 mL reaction solution by mixing ethanol and ferrocene at a concentration of 10 mg/mL and then added it into the injection pump, which was connected to the reactor inlet. Heating the reactor to a reaction temperature of 600 °C, we then injected the reaction solution at a speed of 1 mL/min into the reactor for 2 h under the protection of nitrogen. It then cooled to ambient temperature. The ceramic support was taken from the reactor, and the carbon material on the surface of the SiC support was stripped with scotch tape to obtain the HCNTs-SiC membrane material.

### 2.3. Characterization

The HCNTs-SiC composite membrane morphology and microstructure were observed using field emission scanning electron microscopy (FESEM, Hitachi S-4800, Hitachi Limited, Tokyo, Japan) using an Energy Dispersive Spectrometer and a transmission electron microscope (TEM, JEM-2100 Electron Microscope, JEOL Ltd., Tokyo, Japan). The thermal stability of HCNTs in air was measured via thermogravimetric analysis (TG-DSC STA449, Netzsch Co., Ltd., Hanau, Germany). The molecular structure of HCNTs was characterized using a micro-Raman spectrophotometer (LABRAM HR800, Horiba, Kyoto, Japan). The pore size of the composite membrane was measured using a capillary flow porometer (PMI, ipore-1500, Porous Materials Inc., Philadelphia, PA, USA). Water, oil, and oil under water contact angles were measured using a wettability analyzer (DropMeter A-100, Hai Shu Mai Shi, Ningbo, China). The surface roughness and 3D structure were observed using the Surface Profiler (Laser Microscope, Keyence VK-1000, Keyence Corporation, Ōsaka shi, Japan). The chemical groups of the HCNTs were identified using the Fourier transform infrared spectrometer (FTIR, Nicolet 8700, Thermo Nicolet Corporation, Madison, WI, USA). The W/O emulsions were observed using a metallographic microscope (CX40 M, Sunny Optical Technology Co., Ltd., Ningbo, China), and the droplet size distribution of emulsions before and after filtration was measured through subsequent image processing using analysis software (Nano Measurer 1.2). Furthermore, the helical diameter and pitch of the HCNTs were also obtained using Nano Measurer 1.2 based on the SEM images. The way to obtain particle size or diameter through Nano Measurer 1.2 is by opening the SEM or TEM images, setting the reference line according to the scale in the SEM or TEM images, and then marking the distance you want. Finally, it is calculated automatically. It should be noted that all of the error bar data in this article were obtained through tests on different samples under basically the same conditions or different parts of the same sample, and they have relatively rigorous statistical significance. In addition, the corresponding error bars have not affected the relevant data results, but they indeed have great significance for scientific research and exploration.

### 2.4. Separation Performance

The flux (F) and water permeance (P) of the composite membrane were calculated through Equations (1) and (2):

(1) $$F=\frac{V}{A_{0}\times ∆t}$$

(2) $$P=\frac{V}{A_{0}\times ∆t\times ∆p}$$

where *V* (L) is the water volume on the permeate side, *A*_0_ (m^2^) presents the efficiency of the membrane area involved during the filtration process, *∆t* (h) is the filtration time, and *∆p* (bar) is the transmembrane pressure (TMP).

The rejection efficiency (R) of the material was calculated through Equation (3):

(3) $$R=\frac{C_{f}-C_{p}}{C_{f}}$$

where *C_f_* and *C_p_* represent the oil-in-water concentrations (mg/L) on the feed and the filtrate sides, respectively.

The O/W emulsion (500 mg/L) was diluted with water in different proportions (1:1, 1:5, 1:10, 1:15, 1:20), and the correlation curve between O/W emulsion concentration and the UV absorption value was drawn. According to the calibration curve, the concentration of the oil–water emulsion on the feed side and the permeation side was calculated.

## 3. Results and Discussion

### 3.1. Effect of Fabrication Parameters on HCNTs’ Growth

**Figure 2:**
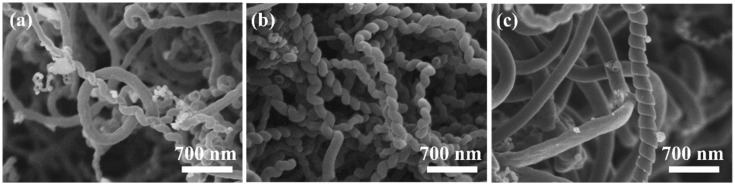
SEM images of the CVD products prepared at different CVD temperatures: (**a**) 580 °C; (**b**) 600 °C; and (**c**) 620 °C.

CVD temperature was the key factor affecting carbon nanotubes’ growth. As shown in Figure 2a–c, helical carbon nanotubes began to appear, but only a few HCNTs could be observed at 580 °C. When the temperature was 600 °C, the CVD products were mainly HCNTs with uniform diameter and regular pitch. When the temperature increased to 620 °C, carbon materials prepared using the CVD consisted of many straight carbon nanotubes and few HCNTs with small pitch.

**Figure 3:**
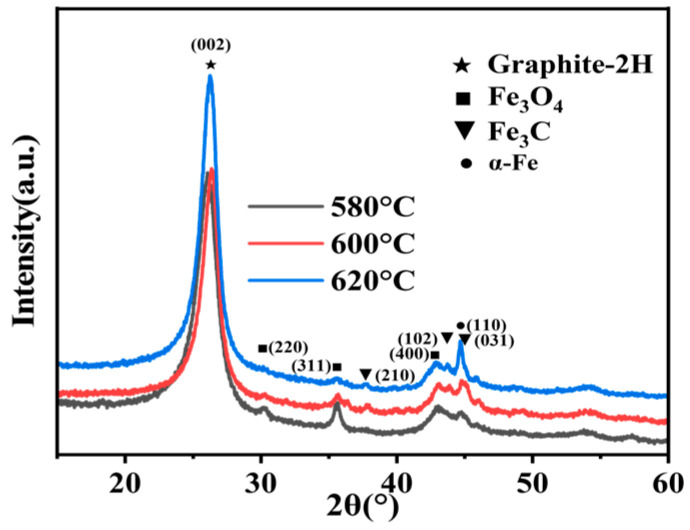
XRD pattern of the CNTs prepared at different temperature.

It is well known that the microscopic morphology of CNTs is determined by the catalyst. In this work, the reactants were ferrocene and ethanol. The former provided the catalyst required for the growth of CNTs, while the latter supplied the carbon atomic groups for CNTs’ formation. In the XRD pattern of the three samples (Figure 3), no characteristic peak of ferrocene (JCPDS 29-1711) appeared, but the peaks of α-Fe (JCPDS 06-0696) and Fe_3_C (JCPDS 35-0772) emerged. This indicated that ferrocene pyrolyzed at a high temperature, generating α-Fe and Fe_3_C, which played the role of CNTs’ growth catalyst. At 580 °C, the catalyst was mainly in the form of α-Fe, whose (110) and (211) planes had different binding energies, and the HCNTs began to form. When the temperature increased to 600 °C, the characteristic peak of α-Fe still existed, and peaks at 37.7°, 43.7 °, and 45.0° began to appear. These peaks were associated with (210), (102), and (031) crystalline planes of the Fe_3_C phase. This was attributed to the temperature increasing, and, as the diffusion of carbon in iron increased, it resulted in the accelerated transformation of α-Fe to Fe_3_C, which could promote rapid graphite precipitation. Therefore, carbon nanotubes mainly exhibited helical structures. When the temperature was higher, iron atoms quickly saturated with carbon, forming Fe_3_C, which was the typical CNTs catalyst. However, it should be noted that the carbon precursor contained the oxygen element, which would react with iron at a high temperature, resulting in the formation of some Fe_3_O_4_ (JCPDS 79-0419) in the samples.

**Figure 4:**
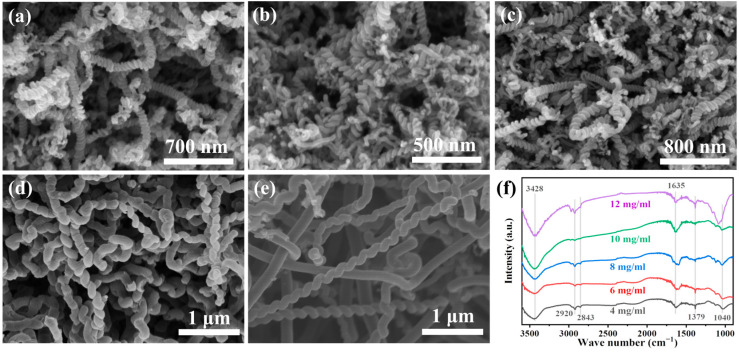
SEM images and (**f**) FTIR spectrum of the HCNTs prepared at different Fe(CP)_2_/C_2_H_5_OH concentrations: (**a**) 4 mg·mL^−1^; (**b**) 6 mg·mL^−1^; (**c**) 8 mg·mL^−1^; (**d**) 10 mg·mL^−1^; and (**e**) 12 mg·mL^−1^.

Figure 4a–e were the SEM images of HCNTs prepared through different reaction solution concentrations. Although helical carbon nanotubes could be observed in all of the sample images, there were significant differences in the helical structures, which indicated that the reaction solution’s concentration had an impact on the microstructure of HCNTs. Figure 4f shows the FTIR spectrum of these samples, where the typical peaks of those HCNTs were similar, indicating that the solution’s concentration only affected the HCNTs’ microstructure but had no obvious influence on the surface chemical groups.

**Figure 5:**
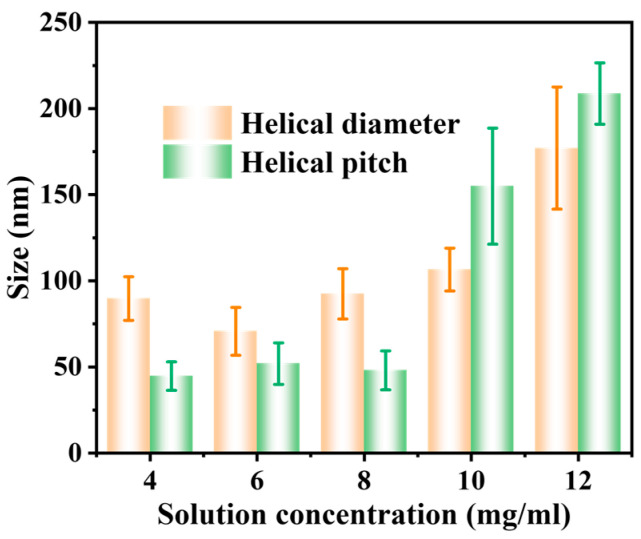
Helical diameter and pitch of the HCNTs prepared using different solution concentrations.

When the concentration of ferrocene/ethanol was 4 mg·mL^−1^, 6 mg·mL^−1^, and 8 mg·mL^−1^, the average pitch of HCNTs was 44.68 ± 8.3 nm, 51.91 ± 12.04 nm, and 47.99 ± 11.3 nm, respectively (Figure 5). This was due to the low concentration of the solution, generating the small catalyst particles, which induced the CNTs to twist with the small angle. At this time, the diameters of HCNTs were 89.72 ± 12.69 nm, 70.68 ± 13.91 nm, and 92.64 ± 14.59 nm, respectively. In addition, when the concentration increased to 10 mg·mL^−1^, the HCNTs’ pitch was 154.91 nm, and the helical structure became orderly (the helical diameter was 106.54 ± 12.42 nm). With the further increase of the concentration to 12 mg·mL^−1^, the pitch increased to 208.69 ± 17.83 nm, but few carbon nanotubes still retained the helix structure. The significant error bars of the helical diameter and pitch data indicated that the carbon materials prepared under the same conditions also had differences, further demonstrating that the particle size of the catalysts prepared by the chemical vapor deposition method was not so uniform. However, these results indicated that the pitch of HCNTs was positively correlated with the amount of ferrocene added, but the dissolution of ferrocene in ethanol tended to be saturated, and ferrocene was more likely to precipitate in the reactor to form large particles of catalyst, which was converted to Fe_3_C and formed straight carbon nanotubes.

### 3.2. Microstructure of HCNTs

**Figure 6:**
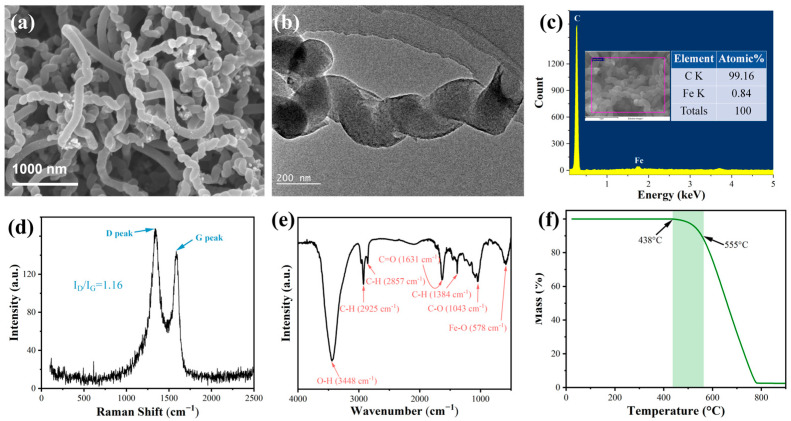
Characterization results of the HCNTs: (**a**) SEM; (**b**) TEM; (**c**) EDS; (**d**) Raman spectrum; (**e**) FTIR pattern; (**f**) TG analysis.

The microstructure of the prepared carbon material is shown in Figure 6. The SEM results (Figure 6a) showed that most of the prepared carbon material under this experimental condition was helical and only a few had a straight structure, indicating the high purity of the helical carbon nanofibers. In addition, it can be seen from the TEM image (Figure 6b) that the prepared helical carbon nanofiber was hollow with open caps, indicating that the prepared fibers were the HCNTs. The elements on HCNTs were Fe (less than 1%) and C (more than 99%), shown in the EDS result (Figure 6c), which meant there was no obvious impurity on it. The I_D_/I_G_ ratios of HCNTs on the surface of SiC were 1.16 (Figure 6d), where the D-peak intensity (I_D_) and G-peak intensity (I_G_) represented the graphitization degree and defect density, respectively. This indicated that there were some structural defects on HCNTs, which could be attributed to the heptagon/pentagon rings in the helix structure. As shown in Figure 6e, the peak observed at 578 cm^−1^ was associated with the Fe–O stretching vibrations due to the presence of Fe_3_O_4_ nanoparticles [44]. Typical peaks at 1034 cm^−1^, 1380 cm^−1^, 2925 cm^−1^, 1631 cm^−1^, and 3448 cm^−1^ were related to C-O vibrations, C-H in-plane bending from -CH_3_, C-H stretch vibration, and the C=O and -OH group from ambient water, respectively. And, the peak at 2857 cm^−1^ was associated with CH_2_ or CH_3_ stretching vibration [45]. These results showed that there were some oxygen-containing groups on the surface of helical carbon nanotubes, indicating structure defects in HCNTs, which was consistent with Raman spectrum results. Moreover, the thermogravimetric curve of HCNTs in air is shown in Figure 6f. It can be seen that when the temperature was higher than 438 °C, the HCNTs began to be oxidized and the mass decreased. The mass loss was 5%, while the temperature reached 555 °C, which meant that the HCNTs also had good thermal stability.

### 3.3. Surface Parameters of HCNTs-SiC Composite Membrane

**Figure 7:**
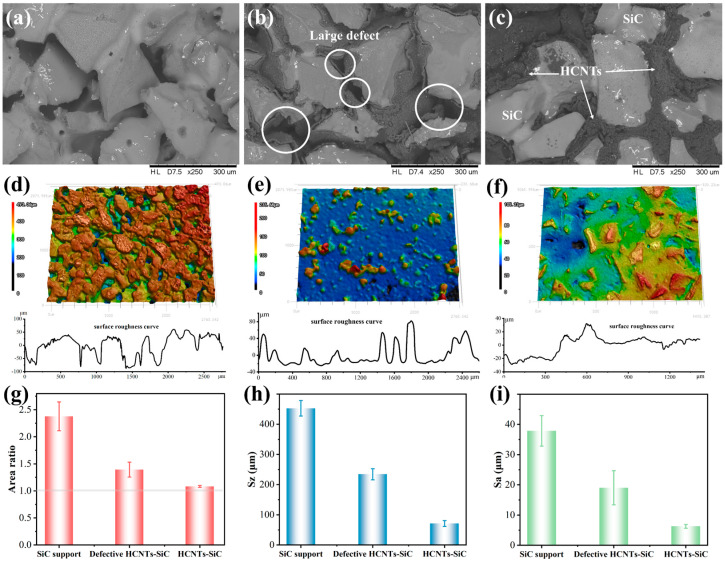
SEM images and 3D morphology of (**a**,**d**) SiC support, (**b**,**e**) defective HCNTs-SiC composite membrane, and (**c**,**f**) HCNTs-SiC composite membrane; (**g**) Sdr, (**h**) Sz, and (**i**) Sa of the three samples.

It was necessary to observe the 3D morphology of the HCNTs-SiC composite membrane because the surface microstructure of the membrane had a significant effect on membrane performance. Sdr represented the increase ratio in surface area of the definition microtopography compared to the projected area. Sa and Sz were the arithmetic mean surface height and maximum surface height of the measured area. The surface microstructures of three samples, the SiC support, the defective HCNTs-SiC membrane, and the ideal HCNTs-SiC composite membrane, were evaluated, as shown in Figure 7. There were obvious particle accumulation pores on the surface of the SiC support (Figure 7a), and these pore sizes and shapes were different. The surface structure of the composite membrane (the defective HCNTs-SiC membrane) prepared with 35 mL reaction solution during the CVD process is shown in Figure 7b. It can be seen that although HCNTs were grown in the pores and modified the channels, some defects still existed, which indicated that the growth amount of HCNTs was insufficient. When the amount of reaction solution increased to 50 mL, the microstructure of the HCNTs-SiC composite membrane is shown in Figure 7c. All of the pores were modified by HCNTs, and no defective pore structure existed. In addition, after adhesive tape treatment, the carbon materials on the surface of the SiC support body were removed, and the SiC particles were exposed, which was consistent with the electron microscopy images in Figure 7b,c.

As can be seen from Figure 7d–i, the porous ceramic carrier formed by the sintering of irregular 200 μm SiC particles had sags and crests on the surface and large roughness fluctuation. The Sz and Sa of the measurement area were 452 ± 26 μm and 38 ± 5 μm. After the growth of HCNTs in the carrier channel, the pore structure was effectively modified, and the “concave area” on the support surface was filled. Therefore, the surface became flat, and the roughness curve fluctuated little. The Sz and Sa of the defective HCNTs-SiC composite membrane and the ideal HCNTs-SiC membrane material were reduced to 234 ± 18 μm and 19 ± 6 μm, 71 ± 9 μm, and 6 ± 1 μm, respectively. In addition, the extended area (surface area) of the materials was analyzed, and we found that the ratio of the extended area to the projected area (Sdr) of the SiC support measured surface was 1.38 ± 0.27, while the Sdr of the defective HCNTs-SiC composite membrane and the ideal HCNTs-SiC membrane were 0.39 ± 0.14 and 0.08 ± 0.02. That indicated that the surface area of the ideal HCNTs-SiC membrane with high flatness was almost the same as its projected area. The presence of HCNTs effectively improved the surface roughness of the support and modified the pore size, which was consistent with the results of the electron microscopy images and the 3D morphology results.

### 3.4. Membrane Structure of HCNTs-SiC Composite Membrane

**Figure 8:**
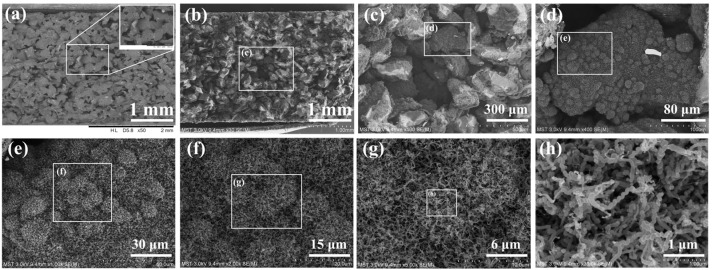
Cross-section SEM images of (**a**) SiC support and (**b**–**h**) HCNT-SiC composite membrane at the same location with different magnification scales.

As seen in Figure 8a, the pore structure of the SiC support was scattered and orderly, and the surface of the 200 μm SiC particles was smooth, with no attachment impurities. This was due to the high-temperature sintering above 1000 °C during the SiC support preparation process, where the activated carbon and adhesive additives were removed. The pore structure of the HCNTs-SiC composite membrane is shown in Figure 8b–h. It was obvious that the helical carbon nanotubes were fully grown in the pore channels of the SiC support, significantly decreasing the pore size of the composite membrane in Figure 8b. The HCNTs were attached to the surface of 200 μm SiC particles and formed a regular bulge of spherical peaks in Figure 8c–e. This could be attributed to the unique three-dimensional interlocking formed by the accumulation of helical carbon nanotubes, which was not found before in our previous work of interweaving and vertical CNT arrays. Furthermore, as evidenced by Figure 8f–h, the carbon nanotubes in the composite membrane fabricated using airflow-induced chemical vapor deposition technology had a helical structure based on continuous magnification observation at the same location.

**Figure 9:**
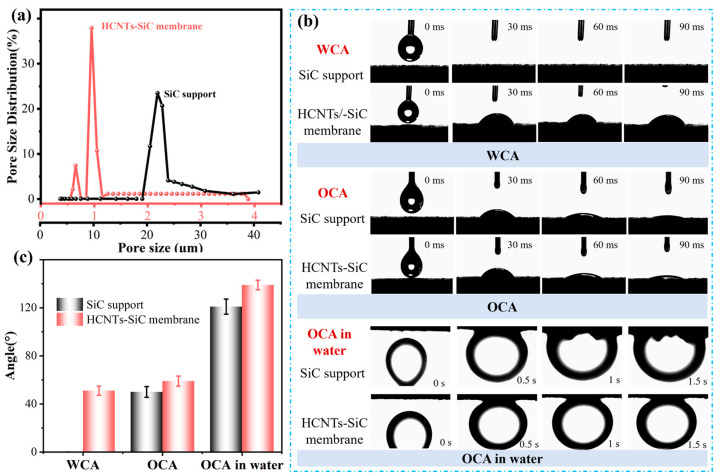
(**a**) Pore size distribution, (**b**) WCA, OCA, and OCA in water images, and (**c**) WCA, OCA, and OCA in water of the SiC support HCNT-SiC composite membrane.

The wettability and pore size of the membrane have a significant influence on the separation performance of the emulsion. As shown in Figure 9a, the average pore size of the SiC support was 21.9 μm, with wide pore size distribution. This was attributed to the fact that irregular SiC particles with average particle sizes of 200 μm and activated carbon powder were difficult to disperse evenly during the pressing and molding process. The water contact angle (WCA) and oil contact angle (OCA) change of the SiC support are shown in Figure 9b. In less than 30 ms contact time, the water droplet was quickly wetted on the surface of the SiC support and permeated into the pore channels. Meanwhile, the oil droplet spread out on the SiC support surface and penetrated into the pore, and the oil contact angle decreased from 50° to 12° (Figure 9c) in 90 ms. In addition, the evolution process of OCA in water of the support can be seen in Figure 9b. The initial OCA in water of the support was 121°, showing oleophobic properties in water. However, as time went by (1.5 s later), the oil droplet was deformed and began to penetrate into the pore. Under the action of surface tension, oil droplets in the air, water droplets, and oil droplets in water eventually diffused into the large support pore. Due to some oxygen-containing groups (SiC particles would generate SiO_2_ during the sintering process at high temperatures) on the SiC support surface and the differences in surface tension and viscosity, water droplets in the air penetrated faster than oil droplets.

The pore size distribution of the HCNTs-SiC membrane is shown in Figure 9a, showing two peaks of 650 nm and 950 nm. HCNTs grew in SiC support pore channels, which effectively reduced the pore size by an order of magnitude. However, due to the wide aperture distribution of the SiC carrier itself, it resulted in the two peaks. The initial WCA and OCA of the HCNTs-SiC membrane were 51 ± 3.8° and 59 ± 4.2°, which indicated that the membrane was hydrophilic and lipophilic in air. It should be noted that although the oil and water droplets both gradually infiltrated into the membrane pores, their penetration rate (Figure 9b) was significantly slower than that on the support. This could be attributed to the combination of the small membrane pore size and the chemical groups of the carbon nanotubes and SiC support. Typical carbon nanotubes are superhydrophobic owing to the only two C-H and C-C bonds. However, besides those chemical bonds, there were some C-O, O-H, and other groups in HCNTs, which led to improvement in the hydrophilicity of the membrane material. The initial value of the underwater oil contact angle of the membrane material was 139 ± 4°, and it did not change with time (1.5 s after), which indicated that the existence of HCNTs made the composite membrane show the characteristics of being hydrophilic and underwater oil phobic.

### 3.5. Separation Performance of the HCNTs-SiC Membrane

**Figure 10:**
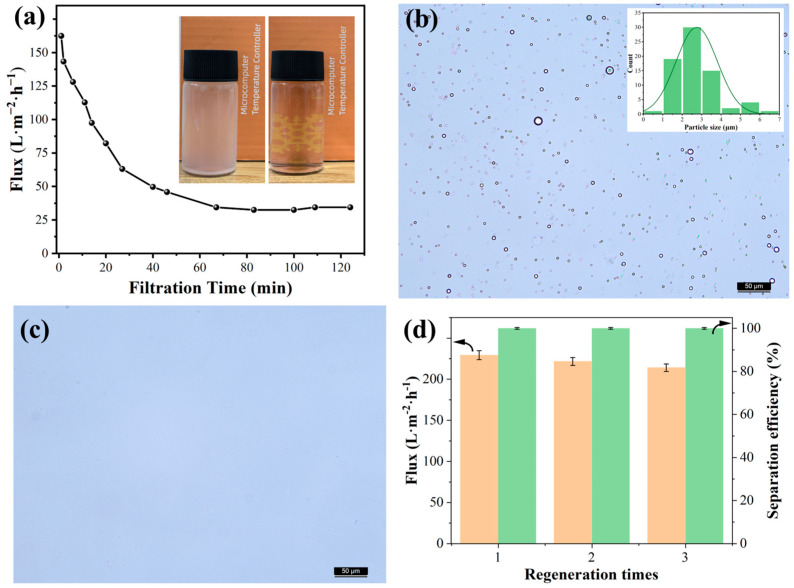
(**a**) Permeation flux of the HCNT-SiC membrane for 500 ppm oil-in-water emulsion and photos (the insert) of the emulsion on the permeating side and the trapping side. (**b**) Micrograph of 500 ppm oil-in-water emulsion and the particles’ size distribution. (**c**) Micrograph of 500 ppm oil-in-water emulsion after filtration. (**d**) Membrane flux and filtration efficiency after regeneration three times.

The separation performance of the HCNTs-SiC membrane for emulsion was investigated through cross-flow filtration. The transmembrane pressure (TMP) was 1.5 MPa, and the emulsion concentration was 500 ppm. In Figure 10a, the membrane flux decayed significantly in the first 30 min and remained relatively stable at 32.48 L·m^−2^·h^−1^ after 60 min. This was due to the fact that oil droplets adhered to the HCNTs’ surface under high TMP, resulting in flux decrease. When the shear force, the transmembrane pressure, and the oil droplet adhesion force were balanced, the flux remained dynamically stable. The particle size of the emulsion droplets was observed before and after filtration, as shown in Figure 10b,c. It could be seen that the particle size of the emulsion stock varied within 7 μm, mainly within 1–3 μm, which could be easily observed. After filtration, it was difficult to find droplets at the same observation scale, which indicated that the membrane material had excellent filtration efficiency for the emulsion, and the inserted optical photo in Figure 10a also verified this. After comparing the UV absorbance of the emulsion, we found that the concentration of the emulsion after filtration was 0.05 ppm, indicating that the separation efficiency of the membrane material was 99.99%. The regeneration ability of the membrane was further investigated using 1.0 wt% NaOH and 0.4 wt% SDBS at 40 °C for 40 min to remove the contaminant emulsion droplets. Through three regeneration experiments (chemical cleaning after filtration for 300 min each time), it was found that the membrane flux slightly decreased from 229.19 ± 5.49 to 214.01 ± 4.48 L·m^−2^·h^−1^ with a stable filtration efficiency of 99.99%, indicating that the membrane had good renewability.

In addition, high requirements for the mechanical strength and stability of membrane materials were needed in oil–water separation. The bending resistance test and the ultrasonic test of membrane materials were carried out to evaluate the mechanical properties and stability. The results are shown in Table 1. It could be seen that the HCNTs had increased the flexural strength from the original 18.8 ± 0.5 MPa to 19.5 ± 0.4 MPa. This was because the presence of HCNTs effectively reduced the porosity of the ceramic support, making the composite membrane become denser. Moreover, the mass of the membrane material slightly decreased after 15 min of ultrasonic treatment, which indicated that the membrane had excellent stability. This was attributed to the fact that the HCNTs with three-dimensional interlocking structures in the ceramic pore channels were not easily damaged.

The recently reported ceramic–CNT composite membranes applied in O/W emulsions’ separation are summarized in Table 2. As it can be seen, the HCNTs-SiC membrane showed good separation performance and excellent stability.

## 4. Conclusions

A composite membrane material was successfully prepared using helical carbon nanotubes in porous silicon carbide pore channels, and its filtration performance for 500 ppm oil-in-water emulsion was investigated in detail. The results showed that the composite membrane had a separation efficiency of 99.99% for emulsions at high TMP (1.5 bar). And, its flux decreased to 32.48 L·m^−2^·h^−1^ after 30 min, which indicated that the hydrophilic and underwater lipophobic interface of HCNTs-SiC membrane could effectively improve the anti-pollution performance in addition to retaining high separation efficiency. In addition, the helical carbon nanotubes have good thermal resistance and grow in twists and turns in pores, and the HCNTs-SiC composite membrane also shows better separation performance for high-temperature oil-in-water emulsions and more cleaning and regeneration methods, such as high-temperature, ultrasonic cleaning. This work provides valuable guidelines for the fabrication of high-performance oil–water separation membranes through inorganic nano-fiber modified porous ceramics.

## Figures and Tables

**Table 1:** Flexural strength and mass changes of HCNTs-SiC membrane.

Sample	Flexural Strength (MPa)	Mass (g)
SiC support without treatment	18.8 ± 0.5	3.21 ± 0.5
HCNTs-SiC without treatment	19.5 ± 0.4	3.66 ± 0.4
HCNTs-SiC after 15 min of ultrasonic treatment	19.5 ± 0.4	3.63 ± 0.5

**Table 2:** Comparison of ceramic–CNT composite membrane for O/W emulsions’ separation.

Sample	Permeance (L·m^−2^·h^−1^)	Oil Rejection (%)	Ref
YSZ with CNT	36	100	[46]
CNTs-Mullite	15.1	100	[30]
HCNTs-SiC	32.48	99.99%	This work

## Data Availability

The original contributions presented in this study are included in the article. Further inquiries can be directed to the corresponding author.
